# Partisan asymmetries in exposure to misinformation

**DOI:** 10.1038/s41598-022-19837-7

**Published:** 2022-09-19

**Authors:** Ashwin Rao, Fred Morstatter, Kristina Lerman

**Affiliations:** 1 Department of Computer Science, University of Southern California, Los Angeles, 90007 USA; 2 Information Sciences Institute, University of Southern California, Marina del Rey, 90292 USA

**Keywords:** Computational science, Statistics

## Abstract

Online misinformation is believed to have contributed to vaccine hesitancy during the Covid-19 pandemic, highlighting concerns about social media’s destabilizing role in public life. Previous research identified a link between political conservatism and sharing misinformation; however, it is not clear how partisanship affects how much misinformation people see online. As a result, we do not know whether partisanship drives exposure to misinformation or people selectively share misinformation despite being exposed to factual content. To address this question, we study Twitter discussions about the Covid-19 pandemic, classifying users along the political and factual spectrum based on the information sources they share. In addition, we quantify exposure through retweet interactions. We uncover partisan asymmetries in the exposure to misinformation: conservatives are more likely to see and share misinformation, and while users’ connections expose them to ideologically congruent content, the interactions between political and factual dimensions create conditions for the highly polarized users—hardline conservatives and liberals—to amplify misinformation. Overall, however, misinformation receives less attention than factual content and political moderates, the bulk of users in our sample, help filter out misinformation. Identifying the extent of polarization and how political ideology exacerbates misinformation can help public health experts and policy makers improve their messaging.

## Introduction

Social media has become the main source of news for a large portion of the population^1^, raising concerns about the quality and reliability of information shared online. These concerns have only grown in urgency with the emerging evidence that social media enabled the spread of misinformation and politically polarized content about the Covid-19 pandemic, its toll, mitigation measures, and the efficacy of interventions, therapies and vaccines^2,3^. According to a Pew Report^4^, political ideology explains a partisan divide in attitudes about Covid-19 and compliance with health guidelines^5^, and there is evidence that misinformation has contributed to vaccine hesitancy in the US, particularly in the politically conservative communities^6^. Since effective response to the pandemic requires collective action, e.g., mass vaccination to achieve herd immunity, social media can exacerbate public health impacts of the pandemic by deepening societal divisions and amplifying health misinformation^7–9^.

Researchers have examined how misinformation and “fake news” are shared online^10,11^, focusing on methods to automatically recognize misinformation^12^ and characterize people who spread it^13^. Social psychologists identified individual psychological traits linked to susceptibility to misinformation: specifically, lack of relevant knowledge^14^ or emotional reliance^15^, as well as religious fundamentalism^16^. By focusing on assessing individual psycho-social characteristics, however, survey-based experiments^14^ do not account for the influence of interpersonal relationships. Peers play an important role in the formation of attitudes and beliefs, including individuals’ perceptions of community’s norms^17^ and their propensity to believe misinformation. For example, discussing climate change with friends and family helped improve acceptance of global warming^18^. People also conform their moral expressions of outrage to those of their peers within social networks^19^. However, the structure of social connections can distort perceptions of social norms^17^, making it all the more important to quantify exposure to misinformation through social networks.

*Polarization* describes the divergence of opinions along an ideological dimension, dividing a population into two groups with sharply contrasting opinions or beliefs^20,21^. Ideology and social networks interact: people seek out online contacts who share their beliefs^22^, following and retweeting social media accounts with similar ideology^23,24^. These interactions facilitate the formation of “echo chambers”, which surround people with like-minded peers who confirm their pre-existing beliefs, thereby amplifying polarization. While studies have demonstrated the existence of partisan echo chambers^2,25–27^, their role in exposing people to misinformation has not been fully characterized.

Existing methods to quantify exposure consider content shared by an individual’s friends. However, at a time when recommendation engines control user engagement, it is critical to consider content external to friendships. Not doing so puts analyses at the risk of under-estimating exposures. Individuals on Twitter can retweet content generated by accounts irrespective of whether or not they have a follow relationship. Prior to retweeting their content, individuals are certainly exposed to it.

To capture some of the complexity of polarization we project it on a two-dimensional space, with axes representing partisanship and factuality (or reliability) of information. Previous works have identified a link between these dimensions: politically conservative social media users are more likely to share misinformation^10,27^ and anti-science content^3^. However, the interaction between partisanship and exposure to misinformation through social connections has not been fully characterized. As a result, we do not know whether partisanship drives selective exposure to misinformation or people selectively share misinformation despite being exposed to diverse and reliable information sources. We organize our research around the following questions:

**RQ1**: How does the polarization of information (along the dimensions of partisanship and factuality) that people *see* compare to the polarization of information that people *share* online? (I.e., are echo chambers two-dimensional?)
**RQ2**: How correlated are the dimensions of polarization, i.e., how much does partisanship correlate with factuality?
**RQ3**: Is there a partisan asymmetry in the exposure to misinformation?
**RQ4**: Do partisans amplify misinformation? Is there a partisan asymmetry in the selective amplification or filtering of misinformation?
**RQ5**: Does factual content or misinformation receive more attention?

Our study addresses these questions by examining online discussions about the Covid-19 pandemic. First, we classify social media users ideologically along political and factual dimensions, assigning them a two-dimensional polarization score. Next, we quantify the polarization of the information users see in their friends’ posts. As a proxy of friends, i.e., accounts users follow, we take accounts users retweet. We identify two-dimensional echo chambers that expose users to ideologically congruent information along political and factual dimensions. However, while social media users tend to surround themselves with peers who share similar views, there are partisan asymmetries in exposure to misinformation. Additionally, the substantial interaction between the two dimensions, also observed in earlier studies^10^, creates conditions for ideologically polarized users to amplify misinformation. These polarized users, who represent hardline partisans on both sides of the political spectrum, selectively share misinformation. However, such users receive less attention than those sharing factual content, and political moderates, who represent the bulk of users in our study, help filter out misinformation, reducing the amount of unreliable content in the information ecosystem. Our study contributes to the understanding of factors shaping public’s exposure to polarized information and misinformation, which could aid public health experts and policy makers in crafting messaging to facilitate consensus and compliance with public health measures.

## Results

We study polarization of online discussions about the Covid-19 pandemic, leveraging the data set of over 260M Covid-19 related tweets between January 21, 2020 and July 31, 2020 to characterize the relationship between information individuals see friends share online, i.e., their *information exposure*, and information individuals themselves *share*.

**Figure 1:**
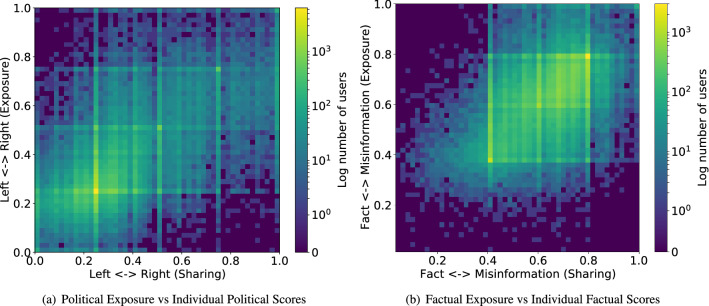
Two-dimensional echo chambers. Heatmap of user polarization scores shows the polarization of information users see and share along (**a**) political (Pearson’s correlation $r=0.61$, $p<0.001$) and (**b**) factual ($r=0.50$, $p<0.001$) dimensions. Colors indicate the number of users given polarization scores.

### Polarization is two-dimensional

We quantify the ideology of information along the dimensions of partisanship and factuality, extracting Pay-Level Domains (PLDs) from URLs embedded in tweets and mapping them to their political and factual scores (see “Methods”). In order to quantify exposures, we leverage interactions in the retweet network and extract PLDs shared by individuals who have been retweeted by the user (see “Methods”). Figure 1 shows the joint distribution of the partisanship (Fig. 1a) and factuality (Fig. 1b) of the information users see friends in their retweet neighborhood share and the information they themselves share. The high density along the diagonal suggests the existence of echo chambers: many users are linked to friends who expose them to ideologically similar information. The correlation between individual ideology and exposure ideology along the partisanship and factuality dimensions are $0.61\ (p<0.001)$ and $0.50\ (p<0.001)$ respectively. There are no partisan asymmetries in the political echo chambers (Fig. 1a), as both liberal and conservative users are exposed to a similar variety of political content. There is some asymmetry in the factual echo chambers (Fig. 1b), since there is much lower density of users in the misinformation bubble. Unlike previous works, e.g.,^25^, the echo chambers we observe are more diffuse, with users linked to friends with more variable ideologies. This is because previous works calculate the average polarization of friends, which gives equal weight to friends who share a lot or a little information, while we aggregate messages shared by all friends when measuring the ideology of exposure.

**Figure 2:**
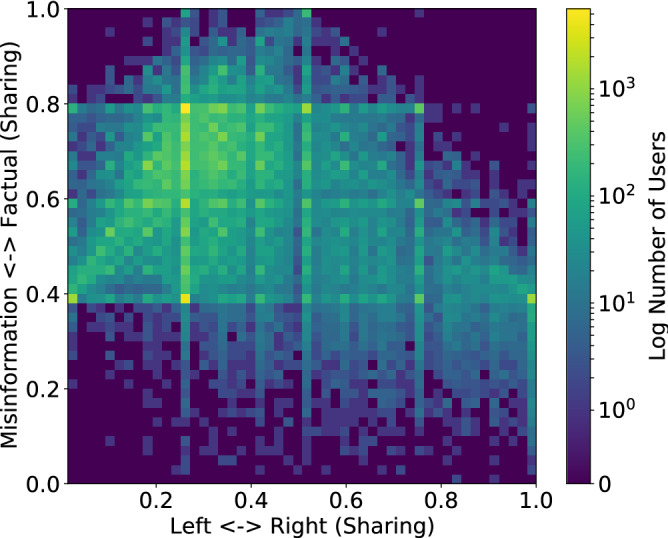
Relationship between dimensions of polarization. Color represents number of users given polarization scores along the political and factual dimensions (Pearson’s correlation $r=-0.198\ (p<0.001)$).

Previous research has identified an interaction between political polarization and misinformation: conservatives share misinformation to a greater degree than liberals^10,11,27^, and they also tend to share more anti-science sources^3^. Our results are consistent with these findings. Figure 2 shows the distribution of user scores in the political-factual space. There is a strong negative correlation ($-0.198,\ p<0.001$) between the two dimensions: users sharing more conservative domains are more likely to share misinformation. However, the large variance masks more nuanced positions. For example, the bright line in the upper-left quadrant shows a phenomenon also observed by^27^ that more extreme liberals have a greater propensity to share misinformation. This shows that polarization amplifies misinformation, a finding we explore in more depth below.

Supplementary Fig. S1 (Refer Supplementary file) contrasts popular topics (hashtags) discussed by people sharing factual information and misinformation. While factual people post messages on health topics (“pandemic”, “wearamask”, “stayhome”), people sharing misinformation are preoccupied with politics (“trump2020”, “kag2020”, “democrats”, “maga”) and conspiracies (“plandemic”, “qanon”, “wwg1wga”). Interestingly, these users also mention media to a much greater extent, using topics like “foxnews”, “7news”, “foxandfriends”, “morningjoe”, and “fakenews”. This may suggest the greater role that media plays in agenda-setting for people vulnerable to misinformation. Also, unlike factual users, people spreading misinformation also discuss unproven cures, like “hydroxychloroquine”.

### Partisan asymmetries in exposure to misinformation

**Figure 3:**
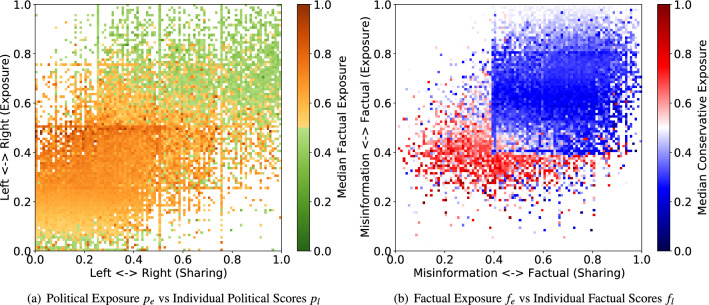
Exposure within echo chambers. (**a**) Color indicates the median factual exposure in each bin. In general, as users share more conservative content while being exposed to more conservative content, they also see more misinformation. Liberal users who are exposed to extreme liberal content also see more misinformation. (**b**) Color indicates the median political polarization score in each bin. Generally, as users generate more misinformation while being exposed to low factual content, they have a higher propensity to share conservative content.

How does the interaction between partisanship and factuality affect what information users are exposed to and, in turn, what information they share? Do people effectively filter out misinformation they see by selectively sharing more factual content?

Figure 3 visualizes user exposure to polarized information. The top row shows user exposure to political and factual information as a function of user political (Fig. 3a) and factual (Fig. 3b) scores. Note that while Fig. 3a,b represents users in the same space as in Fig. 1a,b, i.e., a user’s political/factual scores vs the scores of their political/factual exposures, the colors in the latter show density while the colors in the former show their factual and political opinions respectively. There are several regions of interest in Fig. 3a. Liberal users ($p_l < 0.5$) who are exposed to politically moderate content ($p_e \approx 0.5$) see the most factual information (dark orange). Liberals ($p_l < 0.5$) who are exposed to liberal content ($p_e < 0.5$) generally see more factual (orange) information, although as their exposure becomes more partisan, the share of factual content they see dwindles. Those exposed to extreme left content ($p_e \approx 0$) see more misinformation (green hue). As liberals become more exposed to conservative content ($p_e \rightarrow 1$) they see more and more misinformation. The same is not true of conservatives: conservative users ($p_l>0.5$) who are exposed to right-wing information ($p_e>0.5$) tend to see more misinformation; however, as long as they are not too conservative, exposure to liberal information ($p_e<0.5$) allows them to receive more factual information. Unlike liberals, exposure to politically moderate content ($p_e \approx 0.5$) does not promote factual information among conservatives.

Trends within misinformation echo chambers (Fig. 3b) tell a similar story. Users who share misinformation ($f_l<0.4$) and are exposed to misinformation ($f_e<0.4$) tend to see more conservative content (red), although those who are exposed to more factual content ($f_e \rightarrow 1$) see more liberal information (blue dots). Among people sharing factual information ($f_l>0.6$), those who are exposed to more factual information ($f_e \rightarrow 1$) tend to see politically moderate content (white). The box outline is an artifact of domain polarity scores. MBFC classifies many information sources as “mixed” (0.4), leading to an overabundance of points near that value.

Supplementary Fig. S2 (Refer Supplementary File) visualizes two-dimensional polarization within the echo chambers. Again, the neighborhood exposure vs leaning space is the same as the row above, but the color in each plot shows user polarization or leaning along the alternate dimensions. Supplementary Fig. S2a shows that as partisanship becomes more extreme ($p_l \rightarrow 0$ or $p_l \rightarrow 1$), people are more likely to share misinformation (green). Interestingly, this trend does not strongly depend on partisanship of their exposure ($p_e$). Overall, liberals ($p_l < 0.5$) share more factual information, although those who are more moderate ($p_l \approx 0.5$) tend to share more misinformation (yellow/green) when exposed to more conservative content ($p_e \rightarrow 1$). As shown in Fig. 2b, misinformation-prone users ($f_l<0.4$) tend to post more hardline conservative content (darker red) as they share more misinformation ($f_l \rightarrow 0$) regardless of their exposure; however, those who are most exposed to misinformation ($f_e<0.2$) tend to share more liberal views (blue dots). This is not true for factual users, who tend to share liberal content (blue) regardless of the factuality of their exposure ($f_e$).

### Hardline partisans amplify misinformation

Do people amplify misinformation by selectively sharing fewer factual domains than what they are exposed to?

The off-diagonal elements in the echo chamber plots in Fig. 1 suggest that a sizable fraction of social media users share information that is more polarized and less factual than what they are exposed to, and an equally large number share information that is more factual than what they are exposed to. In other words, some people filter out misinformation from the information ecosystem, while others amplify it. To better understand how the interactions between polarization and misinformation affect how people react to exposure, we define two quantities:

1 $$\begin{aligned} \Delta _f(u)= & {} f_l(u) - f_e(u) \end{aligned}$$

2 $$\begin{aligned} \Delta _p(u)= & {} |p_l(u)-0.5| - |p_e(u)-0.5| \end{aligned}$$

Equation (1) quantifies excess factuality for a given user, i.e., how much more factual content the user shares relative to their exposure. Equation (2) measures excess partisanship, i.e., the relative partisanship of the content the user shares compared to their exposure. Note that we had transformed scores so that instead of partisanship, they measure the degree of political moderacy or extremism regardless of its polarity.

**Figure 4:**
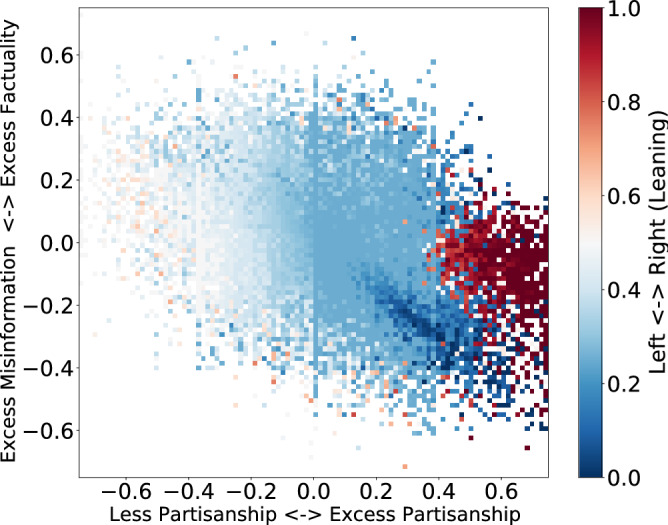
Excess factuality $\Delta _f$ vs excess partisanship $\Delta _p$.

**Table 1:** Results of hypothesis testing for difference in means between the two groups of users along the factuality dimension for various metrics.

Metric $(\theta )$	Factual ($\mu (\theta )_F$)	Misinformation ($\mu (\theta )_M$)	Hypotheses	t-statistic
*T*	38.93	**57.01**	$$H_0:\mu (log(T))_{M} \leq \mu (T)_{log(F)}$$	$${\textbf {16.75}}^{***}$$
			$$\bf H_a:\mu (log(T))_{M} > \mu (log(T))_{F}$$	
*RT*	120.69	**168.28**	$$H_0:\mu (log(RT))_{M}\le \mu (log(RT))_{F}$$	$${\textbf {27.42}}^{***}$$
			$${\textbf {H}}_{{\textbf {a}}}:\mu ({\textbf {log}}({\textbf {RT}}))_{{\textbf {M}}} > \mu ({\textbf {log}}({\textbf {RT}}))_{{\textbf {F}}}$$	
$$A = T+RT$$	159.63	**225.29**	$$H_0:\mu (log(A))_{M}\le \mu (log(A))_{F}$$	$${\textbf {28.89}}^{***}$$
			$${\textbf {H}}_{\textbf {a}}:\mu ({\textbf {log}}({\textbf {A}}))_{{\textbf {M}}} > \mu ({\textbf {log}}({\textbf {A}}))_{{\textbf {F}}}$$	
*R*	**61.97**	48.82	$$H_0:\mu (log(R))_{F}\le \mu (log(R))_{M}$$	$${\textbf {25.64}}^{***}$$
			$$\mathbf{H}_{\mathbf{a}}:\mu (\mathbf{log}(\mathbf{R}))_{\mathbf{F}} > \mu (\mathbf{log}(\mathbf{R}))_{\mathbf{M}}$$	
$$P = R/A$$	**0.57**	0.17	$$H_0:\mu (log(P))_{F}\le \mu (log(P))_{M}$$	$${\textbf {32.86}}^{***}$$
			$${\textbf {H}}_{\textbf {a}}:\mu ({\textbf {log}}({\textbf {P}}))_{{\textbf {F}}} > \mu ({\textbf {log}}({\textbf {P}}))_{{\textbf {M}}}$$	

Significant values are in bold.

Factual users (*F*) have high factuality scores ($f_l \ge 0.6$) while misinformation users (*M*) have low scores ($f_l \le 0.4$). Metrics include: number of tweets (*T*) and retweets (*RT*) generated by the user, the overall activity (*A*), number of times the user is retweeted (*R*) and retweet power (*P*) which is the ratio of number of times retweeted and activity. We performed *t*-tests to assess the statistical significance of difference between the two distributions after log transforming the variables. ***Denotes a statistically significant difference between the means of the two distributions with p-value $<0.001$.

Figure 4 shows the joint distribution of excess partisanship $\Delta _p$ and excess factuality $\Delta _f$. The negative correlation (Pearson’s correlation $r = -0.38$, $p < 0.001$) between the two dimensions suggests that not only do politically hardline social media users (regardless of whether they are liberal or conservative) have a higher propensity for misinformation, but users who amplify politically polarized content also amplify misinformation. The color shows partisanship. Interestingly, both hardline conservatives and hardline liberals are active in amplifying partisanship $\Delta _p>0$ and misinformation $\Delta _f<0$, with liberals playing a more active role in amplifying misinformation. On the other hand, users who are less partisan than their friends ($\Delta _p < 0$) also share more factual information than what they are exposed to ($\Delta _f>0$). By filtering out misinformation, such users play an important role in the information ecosystem. They also tend to be politically moderate.

**Figure 5:**
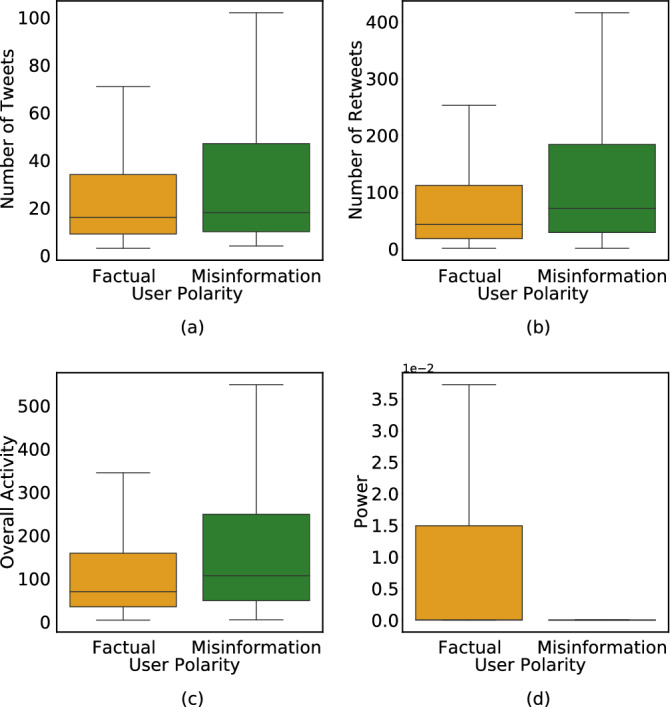
Boxplots comparing activity and power of factual ($f_l \ge 0.6$) and misinformative users ($f_l \le 0.4$). While we notice that misinformative users are more active both in terms of number of tweets and retweets generated, they are retweeted less com- pared to factual users. Subsequently, the ratio of retweets received to overall activity is significantly lower for misinformative users than factual ones.

### Partisan asymmetries in activity

Are users sharing misinformation more active than users sharing more factual content? Does aggressive sharing correlate with more attention? To answer these questions, we define a user’s overall activity as the sum of their tweets *T* and retweets *RT*: $A(u)=T(u)+RT(u)$. To quantify the attention the user *u* receives in response to their activity, we define *retweet power* *P*(*u*) as the ratio of number of times *u* is retweeted *R* and their overall activity:

3 $$\begin{aligned} P(u) = \frac{R(u)}{A(u)} = \frac{R(u)}{[T(u)+RT(u)]} \end{aligned}$$

Boxplots in Fig. 5 visualize the differences in tweet and retweet activity of factual $(f_l \ge 0.6)$ and misinformation $(f_l \le 0.4)$ users. To assess the significance of differences between the two groups, we use the *Student’s* *t-test*. This parametric test of difference between the means of two groups requires the corresponding distributions to be normal. While our metrics (the number of tweets and retweets) have a skewed distribution, taking a log transform increases normality. Table 1 details the null and alternate hypotheses used in our *t*-tests.

From Fig. 5 and Table 1, we see that users who share misinformation tweet and retweet more often and have higher overall activity compared to users who share factual content. Statistically significant *t*-statistics for *T*, *RT*, and *A* in Table 1 reinforce these findings.

Despite their increased overall activity, users sharing misinformation are retweeted less often than factual users $(\mu (R_M) < \mu (R_F))$, significant at $p<0.001$ and have considerably lower retweet power $(\mu (P_M) < \mu (P_F))$ at $p < 0.001$ (Fig. 5d). These findings hint at an increased attention to factual users despite their lower overall activity.

## Discussion

The Covid-19 pandemic exposed societal divisions, with attitudes toward the pandemic and mitigation measures splintering along partisan lines. To study these divisions, we quantified the ideology of information users see and the information they share on social media. Using retweet interactions to quantify exposures, our study gives much needed impetus to consider exposures in the study of online polarization. Although retweets only capture a subset of the follower/friend relationships, they represent who users pay attention to, thereby defining the most important aspect of exposure. A comparison of exposures from follow/friend relationships and retweet interactions is out of scope of this study and provides an avenue for future work.

An important question that arises next is whether we observe echo chambers. Whether individuals share content (original tweets not including retweets) identical in ideological valence to their exposures? Across both dimensions, we find that sharing behaviors are strongly correlated with exposures using Pearson’s correlation metric. Conservatives see conservative content while liberals see liberal content. Similar polarization occurs along the factual dimension. These findings show that echo chambers are two-dimensional.

We then study the relationship between the two dimensions of polarization—political partisanship and propensity for misinformation—and how it asymmetrically affects exposure to misinformation. We find that liberals who are exposed to hardline liberal content see more misinformation, but liberals who are exposed to politically moderate information see more factual content, an effect not seen for conservatives. Conservatives who are exposed to more conservative content are exposed to more misinformation whereas, exposure to liberal content, exposes them to factual information. Moderate liberals share the most factual content irrespective of their exposures whereas, moderate conservatives only do so under liberal exposures. These asymmetries highlight the subtleties of polarization overlooked by previous studies^28–30^.

Lastly, we look at the relationship between partisan extremism and misinformation. We find that highly polarized users, who represent hardline partisans on both sides of the political spectrum, are most likely to amplify partisan content and misinformation. However, such users get less attention than the bulk of users in our study who are political moderates who selectively share more factual content. Therefore, such users filter out misinformation.

There are several limitations to this study worth considering. First, we do not know the actual exposures and thus rely on the retweet network as a proxy. The retweet network provides a subset of relationships in the follower graph. Given that individuals retweet tweets similar to the ones they post themselves, the echo chamber effect inferred by leveraging the retweet network may be overestimated in comparison to the follower network. We have attempted to mitigate the overlap between individual user ideology and retweet exposures by excluding URLs in retweeted content from quantification of the former. Additionally, we also look at all tweets generated by retweeted individuals in our quantification of retweet exposures and do not limit the quantification to tweets that were retweeted. Despite this, using retweet networks may still overestimate echo chamber effects. A natural alternative is the mentions network. It has been shown however, that the mentions network despite allowing individuals to engage in cross-ideological dialogue, may not necessitate individuals to share cross-ideological content with others in their community^28^. This increased heterogeneity of interactions could risk underestimating the echo chamber effect. However, exploring the mentions network as an additional quantification of exposures, one that could mitigate the overestimation of echo chamber effect in the retweet network, remains an interesting avenue for future work. Second, there could be factual/pro-science bias in the data due to the way it was collected. More generally, the keyword-based Twitter crawl used to produce this data could omit nuanced subtopics related to Covid-19 discussions. Lastly, our study focuses on users in the United States. This decision was made because of the United States’ information environment, and due to the dominance of English keywords used to collect the dataset.

This work identifies important differences in the information space of polarized and partisan users. Better understanding of how information is received, and how it propagates, can help public health experts craft more effective messaging. With our work providing quantification for exposures and identifying latent asymmetries, understanding cognitive, social and affective factors driving them can be an interesting avenue for future work. Other important avenues for future work include designing effective interventions for misinformation, assessing the relationship between partisan asymmetries and the binding dimensions of moral thinking such as loyalty, authority and purity, and studying the temporal dynamics of these echo chambers.

## Methods

### Data

In this study, we use the publicly available dataset^31^ comprising of 260.6M tweets related to Covid-19 posted between January 21 and July 31, 2020. These tweets contain at least one of a predetermined set of Covid-19-related keywords (e.g., coronavirus, pandemic, Wuhan, etc.). However, less than 1% of the tweets have geographic coordinates associated with them. We therefore rely on the geolocation method employed in^32^ to determine if the user is within the US. The method works by first extracting the mentions of city or state users frequently have in their profile before employing a fuzzy matching algorithm to match them to their respective states in the US. A manual review of this approach found it to be effective in identifying user’s home state. This leaves us with 48M tweets generated by 2.4M geolocated users in the United States.

### Measuring polarization

We characterize the ideology of information along two dimensions: *partisanship* or *political ideology* and *factuality*. The partisanship dimension captures the source’s political ideology, ranging from hardline liberal to hardline conservative, while the factuality dimension quantifies the source’s predilection for factual content or misinformation. With Media Bias-Fact Check (MBFC)^33^ providing an exhaustive list of media domains and their ideological polarities, previous studies have leveraged individual’s domain sharing behaviors on Twitter^3,25,34^ to quantify ideological alignment. Media Bias-Fact Check lists over 2K pay-level domains (PLDs) under five mutually exclusive categories along the political scale: *Left*, *Center-Left*, *Least-Biased/Center*, *Center-Right* and *Right*. In addition, it also provides a measure of reporting quality for more than 3.5K PLDs along six factuality categories: *Very Low*, *Low*, *Mixed*, *Mostly Factual*, *High* and *Very High*. PLDs generating pro-science content are categorized as *High* or *Very High* while, PLDs sharing conspiracies, questionable or anti-science content are categorized as *Low* or *Very Low* on the factuality scale. Highly partisan news sources such as *foxnews.com, cnn.com, huffpost.com* generally have a chequered quality of reporting and have been listed as *Mixed*. Table 2 refers to the collection of information sources and their ideological biases.

We use *tldextract*^35^ to extract pay-level domains from URLs in tweets. We filter out tweets and retweets containing pay-level domains that are not categorized under either of the two ideological polarities of interest (Table 2).

**Table 2:** Curated pay-level domains and their polarity scores along political and factual dimensions.

Dimension	Polarity	Pay-level domains
Politics	Left (0)	cnn.com, huffpost.com, dailybeast.com,$\ldots$ (350+ PLD s)
	Center-Left (0.25)	aljazeera.com, independent.co.uk, lincolnproject.us $\ldots$ (500+ PLDs)
	Center (0.5)	gallup.com, pewresearch.co.uk, wikipedia.com $\ldots$ (500+ PLDs)
	Center-Right (0.75)	bostonherald.com, chicagotribune.com, wsj.com $\ldots$ (250+ PLDs)
	Right (1)	foxnews.com, gppusa.com, thenationalherald.com $\ldots$ (250+ PLDs)
Factuality	Very Low (0)	counterthink.com, biggovernment.news, vaccines.news $\ldots$ (180+ PLDs)
	Low (0.2)	911truth.org, althealth-works.com, naturalcures.com $\ldots$ (600+ PLDs)
	Mixed (0.4)	breitbart.com, buzzfeed.com, independent.co.uk $\ldots$ (1000+ PLDs)
	Mostly Factual (0.6)	drudgereport.com, washingtonpost.com, bloomberg.com $\ldots$ (200+ PLDs)
	High (0.8)	azcentral.com, bbc.com, nbcnews.com $\ldots$ (1300+ PLDs)
	Very High (1)	nationalacademyofsciences.org, nature.com, bmj.com $\ldots$ (200+ PLDs)

For the political dimension, $\{0,0.25,0.5,0.75,1\}$ represents *Left*, *Center Left*, *Center/Unbiased*, *Center Right* and *Right* sources respectively. Along the factuality or misinformation dimension, *Very Low*, *Low*, *Mixed*, *Mostly Factual*, *High* and *Very High* are quantified as $\{0,0.2,0.4,0.6,0.8,1\}$ in the same order.

We measure the ideology of information individuals share and the information they see friends share by looking at the political and factual scores of the shared domains.

#### Individual ideology

**Figure 6:**
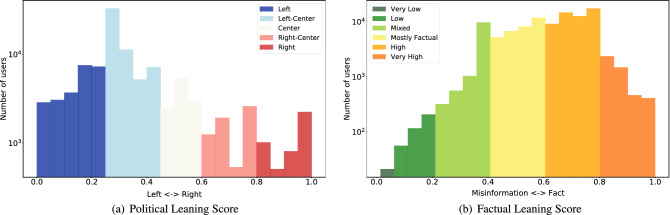
(**a**) Distribution of political leaning domain scores. (**b**) Distribution of factual leaning domain scores.

Similar to previous works^3,25^, we quantify an individual user’s partisanship by averaging over the political scores of the PLDs the user shared. Likewise, we infer individual’s preference for factual information by averaging the factual scores of the PLDs the user shared. This makes our measure of factuality similar to the propensity, or vulnerability, to misinformation used in previous works^10,27^. It is important to note that individual scores quantify the information that users *share* within the online information ecosystem; therefore, users with low factual scores produce more misinformation.

We calculate user *u*’s scores along the political $p_l(u)$ and factual $f_l(u)$ dimensions using Eqs. (4) and (5) respectively. We denote the set of pay-level domains shared by user *u* as *D*(*u*). These include only the domains appearing in *u*’s original tweets (and not retweets). Functions $\Pi (d)$ and $\Phi (d)$ return the political and factual scores of each domain *d*.

4 $$\begin{aligned} p_l(u)= & {} \frac{1}{|D(u)|}\sum _{d \in D(u)} \Pi (d) \end{aligned}$$

5 $$\begin{aligned} f_l(u)= & {} \frac{1}{|D(u)|}\sum _{d \in D(u)} \Phi (d) \end{aligned}$$

Figure 6 shows the distribution of user scores along partisanship and factuality dimensions. The partisanship distribution is skewed to liberal domains, potentially indicating a bias in the Covid-19 data. Similarly, factual scores are skewed towards factuality, and there are relatively few users sharing misinformation or low-factuality content.

#### Ideology of exposure

Understanding polarization people *see* online is challenging for several reasons. On Twitter, as on other social media platforms, users subscribe to accounts of other users to see the content they post. However, the follower graph is usually not available nor is it feasible to reconstruct it from the available APIs. Even when the follower graph is known, the platform’s personalization algorithms may select only a subset of the messages posted by friends, i.e., the accounts the user follows, in the user’s timeline^26^. This can dramatically change the amount and the nature of the information people see^36,37^.

As a proxy of the follower graph, we use the retweet graph, creating links to accounts a user retweets. We consider the retweeted accounts as friends whose activity the user sees. We collect tweets and retweets shared by these friends, extract PLDs and filter out ones that do not have a political or factual scores. In contrast to previous works^25,27,38^, however, which measure ideological polarization of information a user sees to by averaging over friends’ political scores, we aggregate over all tweets posted by friends and calculate political and factual scores of aggregated tweets. This approach factors in the large variation in friend activity: an active friend who posts many messages will have a bigger effect on the user’s information exposure than a less active friend.

Information exposure scores along political ($p_e(u)$) and factual ($f_e(u)$) dimensions are calculated using Eqs. (4) and (5), but now the set of pay-level domains *D*(*u*) corresponds to all domains user *u* sees, which we construct by aggregating over all PLDs shared by *u*’s friends.

**Figure 7:**
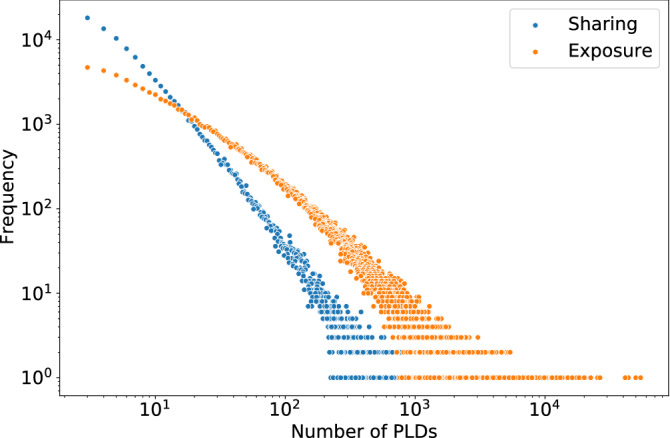
Distribution of number of PLDs users generate (shown in blue) and are exposed to (shown in orange).

After filtering out users who share or see two or fewer PLDs with political and factual scores, we are left with a little over 350K users. Figure 7 shows the distribution of the number of pay-level domains users share in their posts, as well as the distribution of the number of PLDs users see. The difference between the two distributions suggests that some domains are seen much more than they are shared, likely because they are shared by influential accounts with many followers.

Posts retweeted by individuals are ideologically similar to the content they post^39,40^, creating an overlap between ideology and exposure. We mitigate the overlap by (i) not considering PLDs embedded in content retweeted by individuals when quantifying their own ideology, and (ii) when quantifying exposures, considering PLDs in all tweets posted by posted by accounts retweeted by an individual and not just in the content retweeted by the individual. In order to highlight the significance of (i) in mitigating overlap, we run a robustness check that quantifies individual ideology using PLDs in the posts a user tweets and retweets. We find that while results from this robustness check (Refer Supplementary File S1: Accounting retweeted PLDs in quantifying individual ideology) are similar to the ones seen above, we see significant increases in correlation between individual ideology and exposures (Supplementary Figs. S4, S5), as expected. The lower correlations in Figs. 1 and 2 show that removing PLDs embedded in retweets in quantifying individual ideology can mitigate the overlap.

## Supplementary Information

Supplementary Information.

## Data Availability

All datasets and code used to conduct experiments in this study are publicly available in the GitHub repository https://github.com/ashwinshreyas96/Partisan-Asymmetries. Owing to Twitter’s terms of use and service, we are restricted to sharing tweet IDs, which can be hydrated using the Twitter API or third-party hydration APIs. These IDs are accessible in the public repository https://github.com/echen102/COVID-19-TweetIDs.
