# Supplementary material for: Partisan asymmetries in exposure to misinformation
Source: Sci Rep. 2022 Sep 19;12:15671. doi: 10.1038/s41598-022-19837-7 (PMC9484720; doi:10.1038/s41598-022-19837-7)
Supplement: Supplementary file 1 — Supplementary Information. [file 41598_2022_19837_MOESM1_ESM.pdf]

Partisan Asymmetries in Exposure to Misinformation

Ashwin Rao, Fred Morstatter and Kristina Lerman

Topics in factual and misinformative discourse

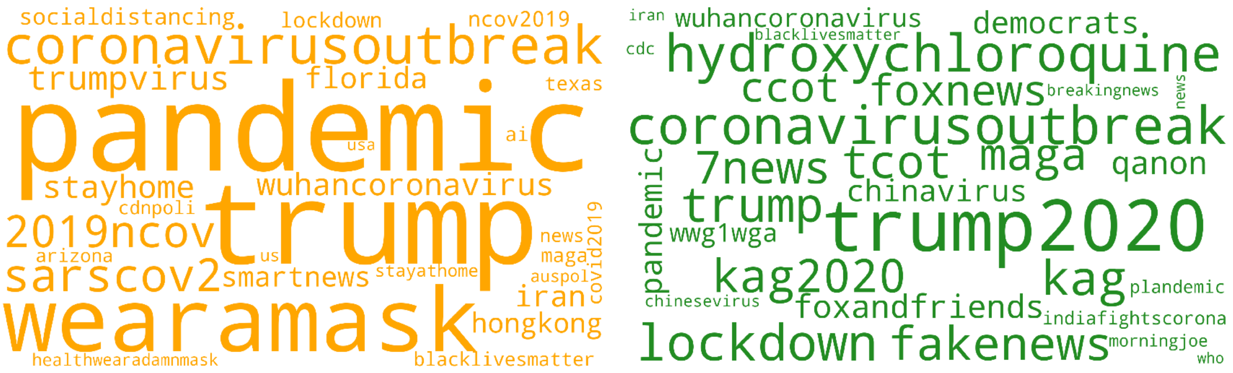

(a) (b)

Supplementary Figure 1: Topics discussed in factual and misinformative discourse. Word cloud of popular hashtags frequently used by (a) factual users ($f_p \geq 0.6$) and (b) users with high propensity for misinformation ($f_p \leq 0.4$).

Ideology within bi-dimensional echo chambers

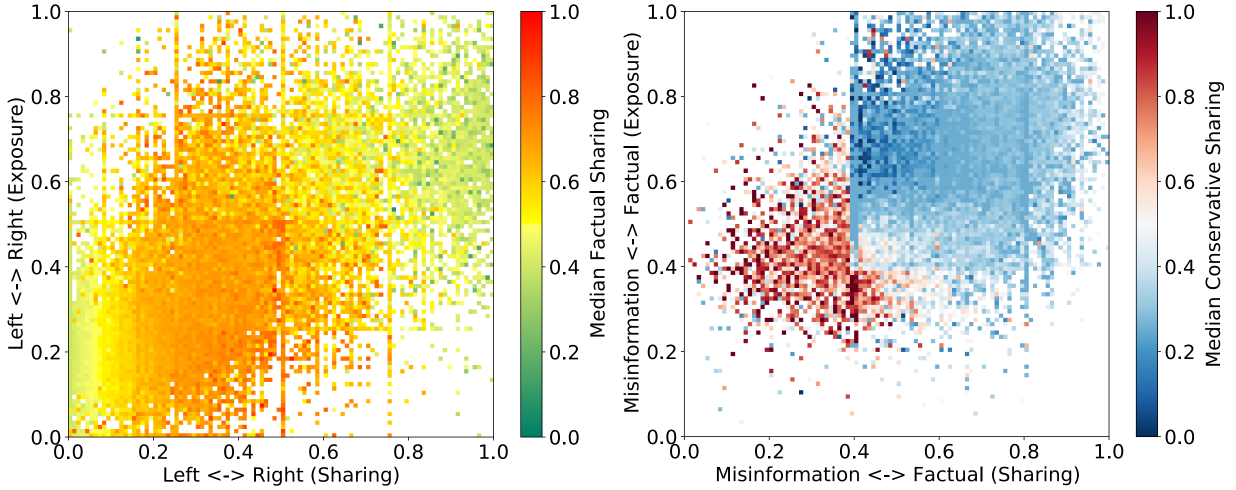

(a) (b)

Supplementary Figure 2: Ideology within bi-dimensional echo chambers. (a) Color indicates the median factual exposure score in each bin. (b) Color indicates the median political exposure score in each bin.

Accounting retweeted PLDs in quantifying individual ideology

In the Methods section we discussed quantifying individual polarization by averaging polarities of PLDs shared by users through their original tweets. People prefer to connect to (e.g., retweet content posted by) others who share their opinions and ideology (boyd, Golder, and Lotan 2010; Metaxas et al. 2015). With retweets symbolizing ideological alignment, we propose an additional measure of individual polarization which includes PLDs from both the user's original tweets and retweets.

We use equations 3 and 4 under Methods to compute the political and factual sharing scores with $D(u)$ denoting the set of PLDs shared by user u through their tweets and retweets. This brings about an overlap between individual shares and exposures as retweeted PLDs now feature in both sharing and exposure sets. This overlap essentially boils down to the duality of a retweet operation. The retweeted tweet factors as "sharing" as the user is disseminating its content to their followers while also exposing the user to its content. Using this method, we are left with over 330K users with both sharing and exposure scores along factual and political dimensions. We reconduct experiments discussed earlier on this score set and find similar trends.

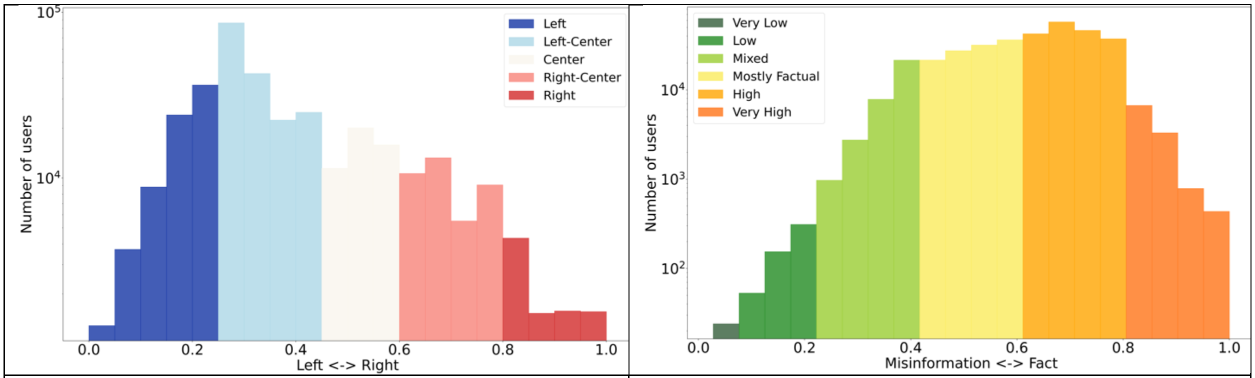

(a) Political Dimension

(b) Factual Dimension

Supplementary Figure 3: Histograms of polarization of information estimated from PLDs in tweets and retweets. Distribution of polarization of information users share along (a) political and (b) factual dimensions.

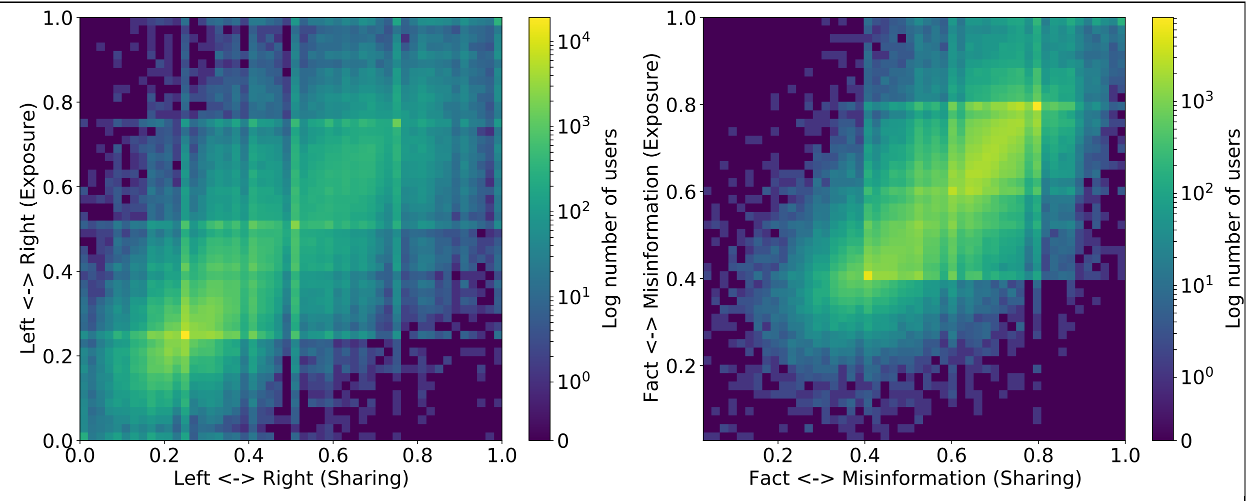

(a) Political Sharing vs Political Exposure (b) Factual Sharing vs Factual Exposure

Supplementary Figure 4. Density of users in ideological echo chambers. Heatmap of (a) political sharing and political exposure ($r = 0.78$, $p<0.001$) and (b) factual sharing and factual exposure ($r = 0.74$, $p<0.001$). Colors indicate the number of users.

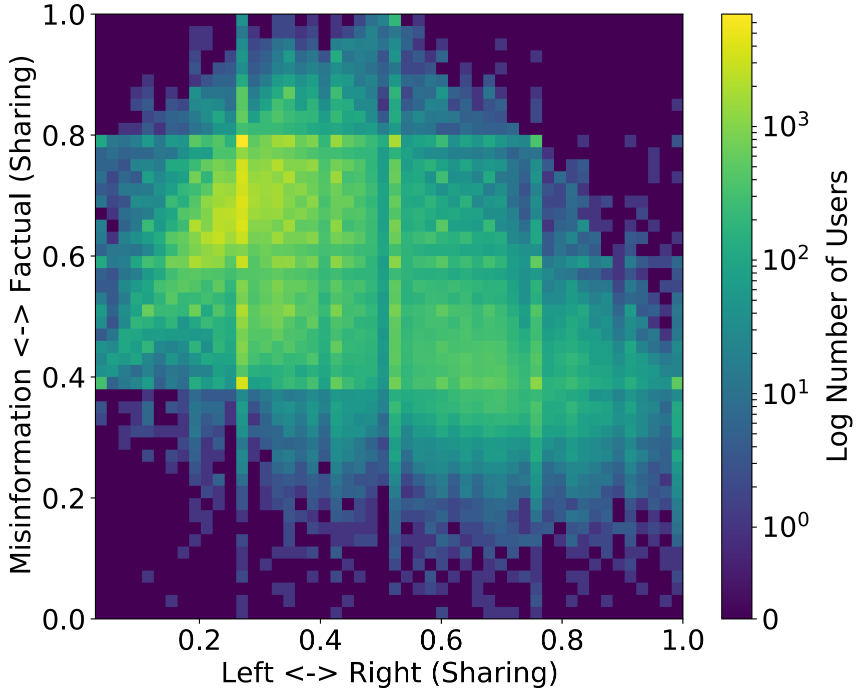

Supplementary Figure 5. Relationship between individual political and factual sharing scores. Color represents number of users. Pearson's correlation $r = -0.446$ ($p < 0.001$).

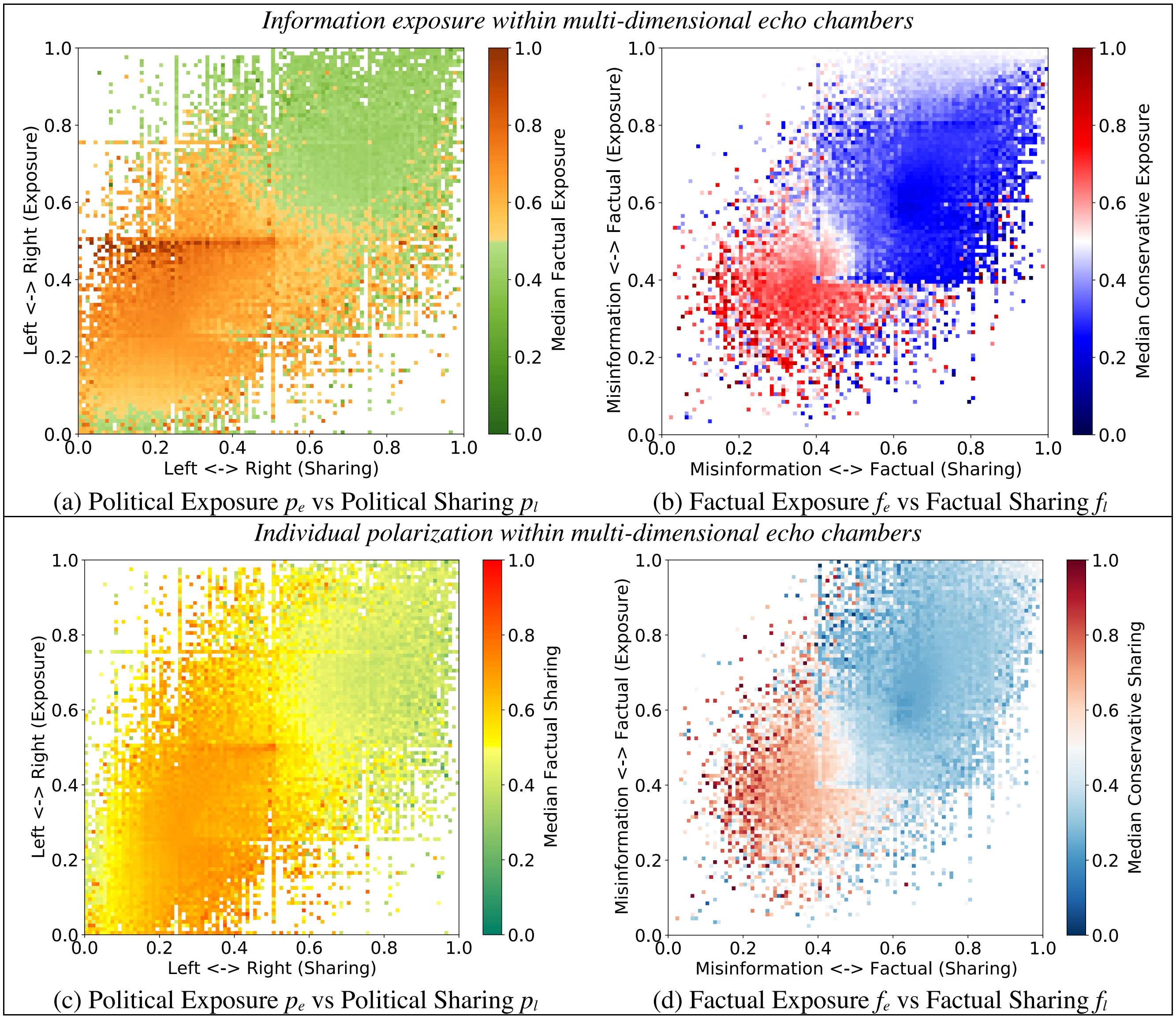

Supplementary Figure 6: Multi-dimensional polarization and information exposure within echo chambers. (a) Color indicates the median factual sharing score in each bin. (b) Color indicates the median political sharing score in each bin.

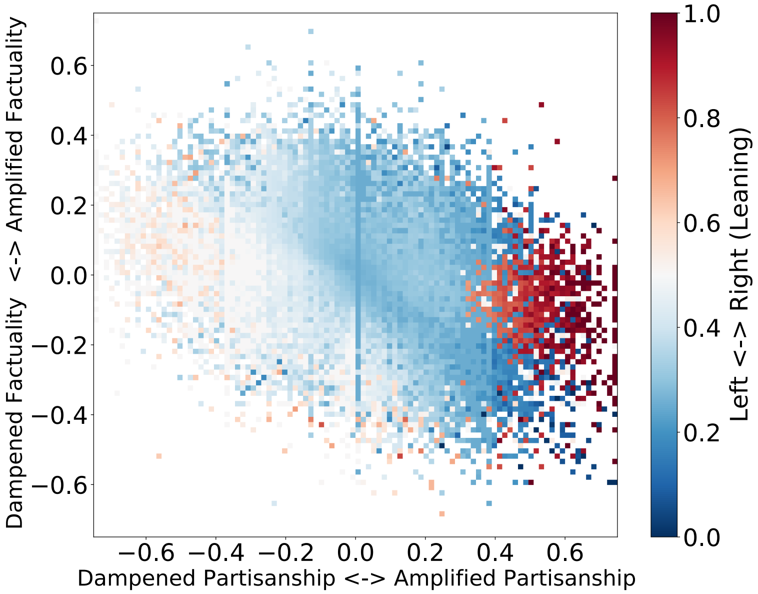

Supplementary Figure 7: Excess factuality Δ_f vs Excess partisanship Δp. Color indicates the political sharing score in each bin.

Metric (θ)	Factual $\mu(\theta)_F$	Misinformative $\mu(\theta)_M$	Hypothesis	t-statistic
T	17.06	20.57	$H_0: \mu(log(T))_M = \mu(log(T))_F$ $H_a: \mu(log(T))_M \neq \mu(log(T))_F$	0.69
RT	100.76	**134.78**	$H_0: \mu(log(RT))_M < \mu(log(RT))_F$ $\boldsymbol{H_a: \mu(log(RT))_M \geq \mu(log(RT))_F}$	**63.19*** **
$A = T + RT$	117.82	**155.36**	$H_0: \mu(log(A))_M < \mu(log(A))_F$ $\boldsymbol{H_a: \mu(log(A))_M \geq \mu(log(A))_F}$	**60.18*** **
R	**24.57**	20.56	$H_0: \mu(log(R))_F < \mu(log(R))_M$ $\boldsymbol{H_a: \mu(log(R))_F \geq \mu(log(R))_M}$	**21.58*** **
$P = R/A$	**0.26**	0.08	$H_0: \mu(log(P))_F < \mu(log(P))_M$ $\boldsymbol{H_a: \mu(log(P))_F \geq \mu(log(P))_M}$	**38.75*** **

Supplementary Table 1. Testing differences between factual & misinformative users for activity & attention parameters Note: Metrics are number of tweets (T) and retweets (RT) generated by the user, the overall activity (A), number times the user is retweeted (R) and retweet power (P) which is the ratio of number of times retweeted and activity. Apart from the comparison of number of tweets T, the results shown here are similar to Table 1 under Findings. We could not identify a statistically significant difference between factual and misinformative users in terms of T. *** denotes a statistically significant difference between the means of the two distributions with p-value < 0.001.

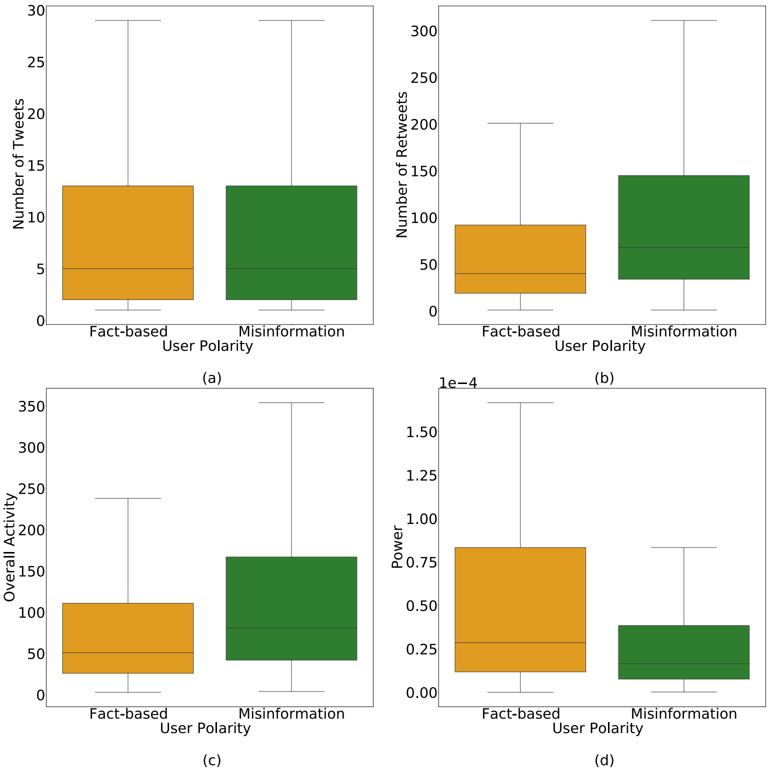

Supplementary Figure 8: Boxplots comparing activity and power of factual ($f_l \geq 0.6$) and misinformative users ($f_l \leq 0.4$). Akin to Figure 5 under Findings, we find that misinformative users despite higher overall activity, garner less attention in comparison to factual users.
